# Methodological optimization for eliciting robust median nerve somatosensory evoked potentials for realtime single trial applications

**DOI:** 10.1088/1741-2552/ae30ac

**Published:** 2026-01-09

**Authors:** Disha Gupta, Jodi Brangaccio, N Jeremy Hill

**Affiliations:** 1 National Center for Adaptive Neurotechnologies, Stratton VA Medical Center, US. Department of Veterans Affairs, Albany, NY, United States of America; 2 Electrical & Computer Engineering Department, State University of Albany, Albany, NY, United States of America

**Keywords:** median nerve stimulation, somatosensory evoked potentials, pulse width, sensory nerve potentials, spinal cord injury, brain computer interfacing

## Abstract

*Objective.* Single-trial measurement of median nerve somatosensory evoked potentials (SEPs) with noninvasive electroencephalography (EEG) is challenging due to low signal-to-noise ratio (SNR), limiting its use in real-time neurorehabilitation applications. We describe and evaluate methodological optimizations for eliciting reliable median nerve SEPs measurable in real time, with reduced reliance on post-processing. *Methods.* In twelve healthy participants, two sessions each, SEPs were assessed at three pulse widths (0.1, 0.5, 1 ms), at a low-frequency stimulation (0.5 Hz ± 10%), and at an intensity sufficient to evoke consistent and robust sensory nerve action potentials and compound muscle action potentials. The evoked potential operant conditioning system platform was used to monitor responses in real time. Feasibility was also evaluated in a participant with incomplete spinal cord injury (iSCI). *Results.* SEP P50 and N70 were reliably elicited in healthy participants, and in individual with iSCI, across all tested pulse widths with minimal discomfort. N70 amplitude increased significantly with pulse width (${\chi ^2}$ = 17.64, *p* = 0.0001, *w* = 0.80), while P50 amplitude remained unchanged. SNR showed a significant pulse width-dependent increase (${\chi ^2}$ = 7.82, *p* = 0.02, *w* = 0.35) with improvements of 40% and 52% at 0.5 and 1 ms, respectively. N70 single-trial separability significantly improved at 1 ms (AUC of 0.83, ${\chi ^2}$ = 8.17, *p*= 0.017), including the iSCI participant (0.84–less impaired hand, 0.79–more impaired hand). Test–retest reliability (intraclass correlation coefficient = 0.70–0.84, *p* < 0.05) was highest at 0.5 ms, indicating more consistent N70 and P50 measurements across sessions at a longer pulse width. *Significance.* Robust median nerve SEPs can be measured at single trials with methodological optimizations such as a longer pulse width (0.5–1 ms), low frequency (0.5 Hz), a consistent afferent excitation guided by nerve and muscle responses, and a robust EEG acquisition system. This setup can be useful for real time SEP-based brain computer interface applications for rehabilitation.

## 1. Introduction

Brain and spinal cord injuries often disrupt somatosensory pathways, critical for sensory-motor coordination (Rosenkranz and Rothwell 2012, Patel *et al* 2014, Matur and Öge 2017, Lo *et al* 2021). The severity of the sensory impairments correlates with motor dysfunction (Scalha *et al* 2011, Meyer *et al* 2014, Gupta *et al* 2017) and their recovery predicts motor recovery (Bolognini *et al* 2016, Chen *et al* 2018c, Zandvliet *et al* 2020). Despite this, rehabilitation focuses on motor training, and less attention is given to improving sensation (Celnik *et al* 2007, Carey *et al* 2011, Turville *et al* 2019). Existing behavioral sensory interventions are complex, subjective, and low in dosage, or involve prolonged sensory stimulation, resulting in variable outcomes (Ridding *et al* 2001, Dobkin 2003, Veldman *et al* 2015, 2016, Carrico *et al* 2016, Conforto *et al* 2018, Tigra *et al* 2020). Alternatively, a closed-loop approach based on cortical responses offers the possibility of reinforcing afferent pathways by associating brain responses with function from individual trials. Providing real-time feedback on a trial-by-trial basis can tightly couple stimulus and response, as shown by select studies using error potentials (Buttfield *et al* 2006, Chavarriaga *et al* 2014, Ferracuti *et al* 2020, Chiang *et al* 2021, Gomez–Andres *et al* 2024, Park *et al* 2025). This is a form of operant conditioning—an approach that has been shown to yield long-term benefits for rehabilitation in the context of spinal reflex conditioning (Wolpaw 1987, Chen *et al* 2006b, Thompson *et al* 2009, 2013, Thompson and Wolpaw 2021).

SEPs, such as those elicited by electrical stimulation of the median nerve, are a known measure of afferent integrity (Chabot *et al* 1985, Misulis and Spehlman 1994, Schaefer *et al* 2002, Poornima *et al* 2013). Damage to sensory pathways is associated with abnormally small and/or delayed SEPs (Perot and Vera 1982, Chabot *et al* 1985, Picozzi *et al* 1989, Gupta *et al* 2017, Ozdemir and Perez 2018, Hubli *et al* 2019), and known to normalize with functional recovery (Curt and Dietz 1996, Ellaway *et al* 2011).

While conditioning of SEPs is known to be possible (Miltner *et al* 1988), it remains relatively understudied—especially using non-invasive EEG—due to the inherently low SNR and reliability of single trial evoked potentials. Moreover, even in healthy individuals, not every stimulus in a stimuli train may consistently elicit a detectable event related potential (Cecotti and Ries 2017). This issue can be compounded post injury, due to injury-related sequelae (Cui *et al* 2015). Factors such as: ongoing cortical oscillations (Kutas *et al* 1977, Jongsma *et al* 2000); attention-based modulation (Hillyard *et al* 1973); neural refractoriness or synaptic adaptation (Quiroga *et al* 2007, Merchie and Gomot 2023); central gating mechanisms triggered by stimulation induced twitches (Rushton *et al* 1981); transient variability in stimulation efficacy due to fluctuations in nerve excitability, skin impedance, or electrode position; and destructive interference from background EEG activity or other electrical noise are known to affect evoked responses. Consequently, SEPs are traditionally obtained by averaging responses over hundreds of trials (Blankertz *et al* 2011, Luck 2014). Eliciting and recording robust single trial (i.e. every individual trial) SEPs with non-invasive EEG for real time feedback, remains a challenge. The ability to operantly condition SEPs may enable new avenues for rehabilitating somatosensory cortical processing and enhancing functional outcomes.

While post-processing techniques such as blind source separation-based methods (Jung *et al* 2001, Delorme and Makeig 2004, Hu *et al* 2005, 2011, Liu *et al* 2011); deep learning methods (Das *et al* 2025); regularization methods (Lu *et al* 2025); Wavelet transform-based methods (Quiroga and Garcia 2003); phase space reconstruction methods (Effern *et al* 2000); Bayesian inference approaches (Truccolo *et al* 2003, Wu *et al* 2014); Expectation maximization (Chen *et al* 2016d); and canonical decomposition methods (Vanderperren *et al* 2013), can significantly enhance evoked potential SNR (Blankertz *et al* 2011), these approaches still depend on multi-trial data and are computationally intensive, limiting their use in real-time applications. In contrast, we focus on developing methods that elicit robust SEPs in single trials, reducing the reliance on extensive post-processing and denoising.

Standard protocols for median nerve SEP typically use short pulse durations (0.1–0.2 ms), stimulation frequencies of 4–7 Hz, and intensities set at 2–3 times the sensory threshold or at motor threshold (Misulis and Spehlman 1994, Cruccu *et al* 2008). The hand/arm position is usually maintained at rest or is unspecified (Misulis and Spehlman 1994: guidelines). These parameters mainly recruit large-diameter fibers (A*β*) of the dorsal column–medial lemniscus pathway, which transmit vibration, touch, and proprioceptive information. Increasing the stimulus intensity at these parameters can enhance the SEP SNR by depolarizing a greater number of sensory neurons, evoking a more synchronized cortical response. However, this would also induce discomfort due to the recruitment of nociceptive fibers (A*δ*) (Dawson 1956, Whitwam 1976), and can co-stimulate the neighboring ulnar nerve, contaminating the cortical responses.

Recent studies describe methods to improve the robustness of single-trial SEPs specifically in lower limb (tibial nerve) stimulation—such as the use of lower stimulation frequency (⩽1 Hz) at a longer pulse width (∼1 ms), while maintaining afferent excitation monitored via the spinal Hoffman reflex (H-reflex) and compound muscle action potential (CMAP) measurements from the soleus muscle (Gupta *et al* 2025a, 2025b). Some of these methods may also be applicable and beneficial for upper limb (median nerve) stimulation. However, eliciting an H-reflex in the flexor carpi radialis (FCR) muscle—the most studied site for H-reflex assessment in median nerve stimulation—is significantly more challenging, especially at rest, due to the higher sensitivity to physiological and anatomical factors (Mercan and Kuruoğlu 2024), and overlap with the CMAP (Gupta *et al* 2021). These challenges are compounded post-injury, due to the increased H-reflex variability (Schimsheimer *et al* 1988, Bodofsky 1999, Eliaspour *et al* 2009).

To address these issues, we explore methods to improve the SEP SNR while minimizing participant discomfort and reducing reliance on extensive signal averaging and post-processing. These include a combination of: (a) alternative parameter choices—including longer pulse widths (0.5–1 ms), suprathreshold intensities guided by sensory nerve and muscle action potentials, and lower stimulation frequency (0.5 Hz), (b) stabilizing effective afferent excitation—by using sensory nerve action potentials (SNAPs), as a guide, as discussed in few studies (Fukuda *et al* 2007), instead of the H-reflex used in previous studies, and (c) reducing SEP acquisition noise—to enhance SNR. We evaluate these strategies in healthy participants and in a pilot case with incomplete spinal cord injury (iSCI), focusing on improving the reliability of P50 and N70 SEP components measured at the somatosensory cortex. We focus on two mid-latency SEP components- P50 and N70, well-known to be elicited by median nerve stimulation (Chabot *et al* 1985, Allison *et al* 1991, Misulis and Spehlman 1994), at the contralateral primary somatosensory cortex and the secondary somatosensory cortex respectively, generally recorded at the centro-parietal areas of the scalp (Misulis and Spehlman 1994).

## 2. Data and methods

### 2.1. Participants

Twelve healthy adults (8 men, 4 women, age (mean ± std): 54.5 ± 14.8 years) consented to participate in the study. Exclusion criteria comprised a history of neurological disorders, use of neuromodulatory medications, presence of open wounds or known scalp infections, pregnancy, or metal implants, including cardiac pacemakers, cochlear implants and neurostimulators. The study also includes one individual (>18 years of age) with a chronic incomplete thoracic spinal cord injury (T5–T6), with marked sensory motor impairments in the leg and the left hand, relying on a wheelchair for mobility (age and gender is withheld to maintain de-identification). All participants provided written informed consent, and the study protocol was approved by the local ethics institutional review board (IRB) at Stratton VA Medical Center (#1726675 and #1584762).

### 2.2. Experiment setup

**Figure 1.**
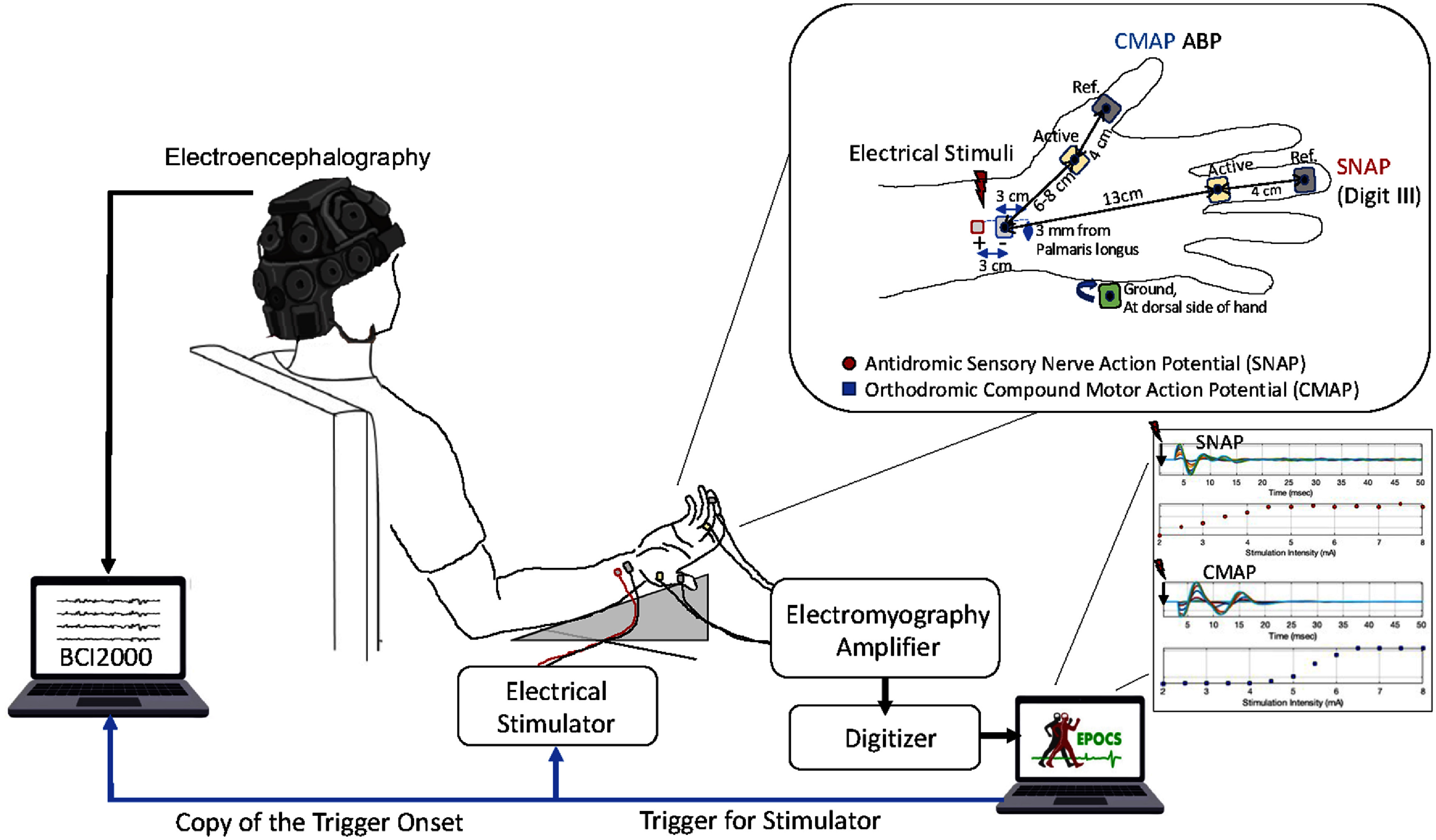
Median nerve electrical stimulation and recording setup for measurement of non-invasive somatosensory evoked potentials (SEPs), antidromic sensory nerve action potentials (SNAPs) and orthodromic compound muscle action potentials (CMAPs). SEPs are acquired with a dry active 19-channel EEG headset (DSI-24), and BCI2000 software. SNAP and CMAP are recorded with an analog amplifier (AMT-8) and a digitizer (NI-6025) and monitored in real time via evoked potential operant conditioning system (EPOCS). Electrical stimulation is delivered at the median nerve with a constant current stimulator (DS8R), triggered by EPOCS. The upper inset shows the stimulation and recording electrode setup. The lower inset shows a representative dataset collected for SNAP and CMAP recruitment curves.

The experiment was setup to evaluate the SEPs elicited by median nerve stimulation at short and long pulse widths (0.1, 0.5 and 1 ms) while controlling for the effective afferent excitation via the SNAP and the direct muscle response (CMAP). The participants were seated upright on a chair with their hands placed at rest on a table in front of them. The stimulation and recording setup on the hand is illustrated in figure 1. Stimulation was applied at the wrist antidromically using a constant current stimulator (DS8R, Digitimer Ltd.), and the recording of the SNAP was performed at the 3rd digit (middle finger), and the CMAP at the abductor pollicis brevis (APB) muscle, using an EMG acquisition system (AMT-8, Bortec Biomedical Ltd.), and the EPOCS software platform (Hill *et al* 2022). SEPs were recorded with a dry EEG headset (DSI-24, Wearable Sensing) and the BCI2000 software (Schalk *et al* 2004, Mellinger and Schalk *et al* 2007). All hardware was synchronized via transistor–transistor logic (TTL) pulses delivered by a digitizer unit, managed by the EPOCS software platform. The EPOCS software also managed the interstimulus interval (ISI) for the stimulation, adding a uniform distribution of jitter ranging from 1.8 s to 2.2 s, averaging to a stimulation frequency of 0.5 Hz. Data was collected in two separate sessions, where possible, to test the stability of the responses. To maintain consistent positioning of the EEG electrodes across sessions, we used a dry EEG headset with electrodes mechanically pre-fitted to the headset core, ensuring uniform interelectrode distances. The core was positioned as per manufacturer guidelines, with the center of the headset at the intersection of the midline and the line connecting nasion and inion. The headset band was positioned at the middle of the forehead, and the distance between Fz electrode and the band was matched to the distance between Pz electrode and the posterior band. To maintain consistency in the EMG electrode positions, we positioned the electrodes based on precise measurements, as shown in figure 1, along with anatomical landmarks such as the interphalangeal and wrist creases.

Participants were asked to maximally abduct the thumb 3–4 times, to assess their EMG at the maximum voluntary contraction level. A comparatively negligible level of background EMG (resting state) was maintained throughout the experiment. At each session, for each participant and pulse width, first, a few test pulses were delivered to allow the participant to get accustomed to the sensation. This was followed by a recruitment curve for SNAP and CMAP to determine the Target stimulus intensity for that pulse width. The EPOCS software platform was used to visualize the SNAP and the CMAP responses at every trial, along with the formation of the recruitment curve. The EPOCS software was also used to continuously monitor and maintain the background muscle activity in the APB muscle at the resting state, allowing the stimulation only when the background was maintained at a low level for at least 200 msec. At each pulse width, a recruitment curve of SNAP and CMAP was obtained, followed by an Assessment run.

### 2.3. Peripheral stimulation

Peripheral stimulation was applied to the median nerve at the wrist using a constant-current electrical stimulator (DS8R, Digitimer Ltd.). Monophasic pulses were delivered via surface electrodes (Vermont Medical Inc.), placed antidromically (Kiernan *et al* 2004, Kuwabara *et al* 2006) at the wrist between the palmaris longus and the FCR tendons. The cathode (22 × 35 mm electrode) was positioned approximately 3 cm proximal to the distal wrist crease, while the anode (22 × 22 mm electrode) was placed 3 cm proximal to the cathode. The stimulating electrodes are placed longitudinally to avoid concomitant stimulation of the ulnar or radial nerves (Valls-Sole *et al* 2016). Stimulation was delivered at an average frequency of 0.5 Hz, i.e. at 2 s ISI with a jitter of up to 10% of the ISI (i.e. 1.8–2.2 s). We evaluated the effect of three different pulse widths: 0.1 ms, 0.5 ms, and 1 ms. The target stimulus intensity was determined for each pulse width and for each participant via a recruitment curve at that pulse width.

*Recruitment Curves* for SNAP and CMAP were acquired by measuring these potentials at gradually increasing stimulus intensity in steps of 0.5 mA. Four trials were acquired at each intensity. The participants were asked to actively indicate any onset of discomfort or pain, whereby we limited further increase in stimulation intensity at that pulse width. The recruitment curves were instantaneously analyzed in EPOCS software. The smallest intensity that elicited a consistently discernable SNAP (i.e. more than three times the standard deviation of background responses, in at least 3 out of 4 trials), along with a consistently small but discernable CMAP was determined as the stimulation intensity for the subsequent assessment run. The SNAP at this intensity was referred to as the Target SNAP response, and the CMAP as the Target CMAP.

*Assessment Run* was performed at the selected stimulation intensity for at most 70 trials, with other stimulation parameter settings remaining unchanged. The SNAP and the CMAP were continuously monitored, with the aim to maintain the CMAP within ±20% of the Target CMAP, which maintained the SNAP within ±20% of the Target SNAP, to maintain the effective afferent stimulation across trials. SNAP and CMAP should follow each other; one could monitor SNAP in this role instead as well–this remains to be explored and may be best performed with automated monitoring and intensity adaptation. The stimulus intensity could be increased or decreased in steps of 0.1 mA during the run. If the stimulus intensity was changed, it was kept at that level unless/until another change was required. The charge delivered at each pulse width was calculated as:

\begin{equation*}{\text{ Charge }}\left( C \right) = {\text{Pulse width }}\left( {{\text{sec}}} \right){ }x{\text{ Current }}\left( A \right).\end{equation*}

### 2.4. EMG recording

Surface bipolar EMG was recorded using an 8-channel analog amplifier (AMT-8, Bortec Biomedical Ltd, Canada) and a pre-amplifier (×500). Self-adhesive snap electrodes (22 × 22 mm, Ag/AgCl, Vermont Medical Inc.) were used for recording. SNAP was recorded in an antidromic setting as per nerve conduction study recommendations (Buschbacher 1999, Chen *et al* 2016a), with the active and reference electrodes placed on the finger pad and third phalanx of the middle finger (illustrated in figure 1). These were kept 3 cm apart (center-to-center) (Walker 1996). The ground was positioned on the ulnar side of the back of the hand. The direct muscle response was measured at the APB muscle of the same hand. The active electrode was placed at the thenar eminence (fleshy part at the base of the thumb), with a reference at the proximal phalanx. The distance between the active electrode at the finger and the stimulation point at the wrist was 13 cm.

Care was taken to maintain a neutral resting position (Cuevas-Trisan and Ojeda-Rodriguez 2006), with the elbow relaxed and bent at 90° beside the upper body, forearm supinated and resting on a small positioning wedge or cushion, for comfort and to maintain the wrist at slight flexion (approximately 15–30°), with APB at rest.

The EMG data was bandpass filtered at the hardware level between 10–1000 Hz and digitized by an analog-to-digital converter (PCIe-6321, National Instruments), at a sampling rate of 3200 Hz.

The EMG data were continuously transmitted to the EPOCS software in real time, which monitored the background EMG and triggered the stimulator at the predefined ISI, provided that the background EMG remained at a predefined resting state for 200 ms. The EPOCS software also rendered the EMG response elicited at the APB and the SNAP elicited at the middle finger, in real time.

### 2.5. SNAP and CMAP analysis

The nerve potential data and the EMG data were band-pass filtered between 10–1000 Hz by the amplifier, which adequately removed any movement artifacts. Data was further pre-processed and band-pass filtered (100–300 Hz) during signal processing, to remove line noise artifact, and improve the SNR. Epochs were created from −50 to 50 ms, relative to the stimulation onset at 0 ms, as a SNAP is typically expected at a latency of 3–4 ms (Nashed *et al* 2009) and a CMAP at a latency <20 ms. The SNAP and CMAP responses were calculated as the peak value of the signal within investigator-determined time windows, instead of the peak-to-peak value which can be affected by the inter-electrode distance (Andersen 1985, Evanoff and Buschbacher 2004). Background response was calculated as the median of the rectified epochs of −50 to −15 ms, relative to the stimulation onset at 0 ms. Recruitment curves were obtained by averaging these responses across groups of four trials at each stimulation intensity. For the Assessment runs, the SNAP and CMAP responses were averaged across all the pre-processed trials. A representative SNAP and CMAP is shown in figure 3, which show the expected signal morphology as mentioned in other nerve conduction studies (Valls-Sole *et al* 2016).

### 2.6. EEG recording

19-channel referential EEG was recorded using a non-invasive dry active EEG headset (DSI-24, Wearable Sensing, CA). The electrodes (Fp1, Fp2, Fz, F3, F4, F7, F8, Cz, C3, C4, T7(T3), T8(T4), Pz, P3, P4, P7(T5), P8(T6), O1, O2) were placed according to the international 10–20 EEG system (Klem *et al* 1999), with the ground electrode positioned at FPz and the reference at the linked earlobes. Data were acquired at a sampling rate of 300 Hz using the BCI2000 software (Schalk *et al* 2004, Mellinger and Schalk *et al* 2007). To ensure precise synchronization of EEG and EMG data, electrical stimulus pulses from the EPOCS system to the electrical stimulator were duplicated and transmitted to the EEG acquisition system as 5 V TTL pulses.

### 2.7. EEG data analysis

The multichannel EEG data was preprocessed by removing bad channels, notch filtering (55–65 Hz), and band pass filtering (2–40 Hz) with a zero-phase filter (Butterworth, model order 2). Next, a Laplacian filter was used for spatial filtering, followed by epoching the data from −50 to 300 ms, relative to stimulation onset. A baseline correction was applied by subtracting the mean of a 50 ms baseline per trial. Subsequent processing was performed separately for (a) conventional averaged SEP analysis and (b) single-trial SEP evaluation.

#### 2.7.1. Averaged SEP analysis

**Figure 2.**
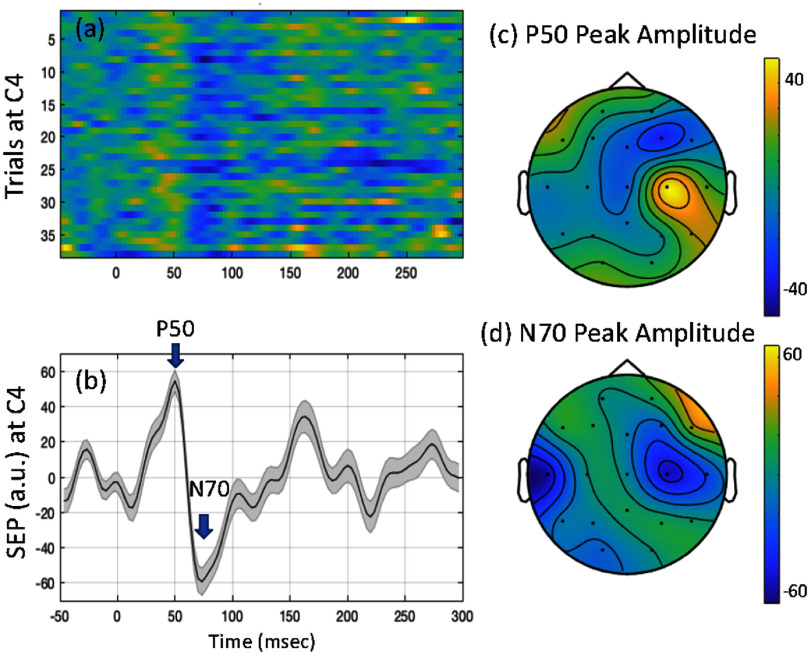
Representative somatosensory evoked potential (SEP) data from one participant: (a) heatmap of trials at contralateral sensorimotor scalp region (C4 EEG electrode for left hand stimulation) (b) SEP (mean and standard error) of the trials shown in (a). Components P50 and N70 are marked. (c) Spatial distribution of the P50 peak shown on a whole head topography (d) spatial distribution of the N70 peak shown on a whole head topography.

To assess the conventional averaged SEP response, the above pre-processing was followed by trial denoising, which involved removal of bad trials identified by trial statistics. SEPs elicited by median nerve stimulation were measured at the contralateral sensorimotor regions i.e. spatially filtered C3 for right hand stimulation and C4 for left hand stimulation, as these are known to be involved in sensory processing at the hand/arm region (Roux *et al* 2018), and as per the sensory homunculus for upper limbs (Penfield and Rasmussen 1950). A representative SEP obtained in this study is shown in figure 2. We focus on the mid-latency positive component (P50) and a following mid-latency negativity (N70). These are known to be evoked in the primary and secondary somatosensory regions, during cortical processing of upper limb sensation (Misulis and Spehlman 1994). The SNR and spatial specificity of the SEP response was assessed, followed by an evaluation of the *r*^2^ across trials to quantify the variance explained by the stimuli response and background activity.

**Figure 3.**
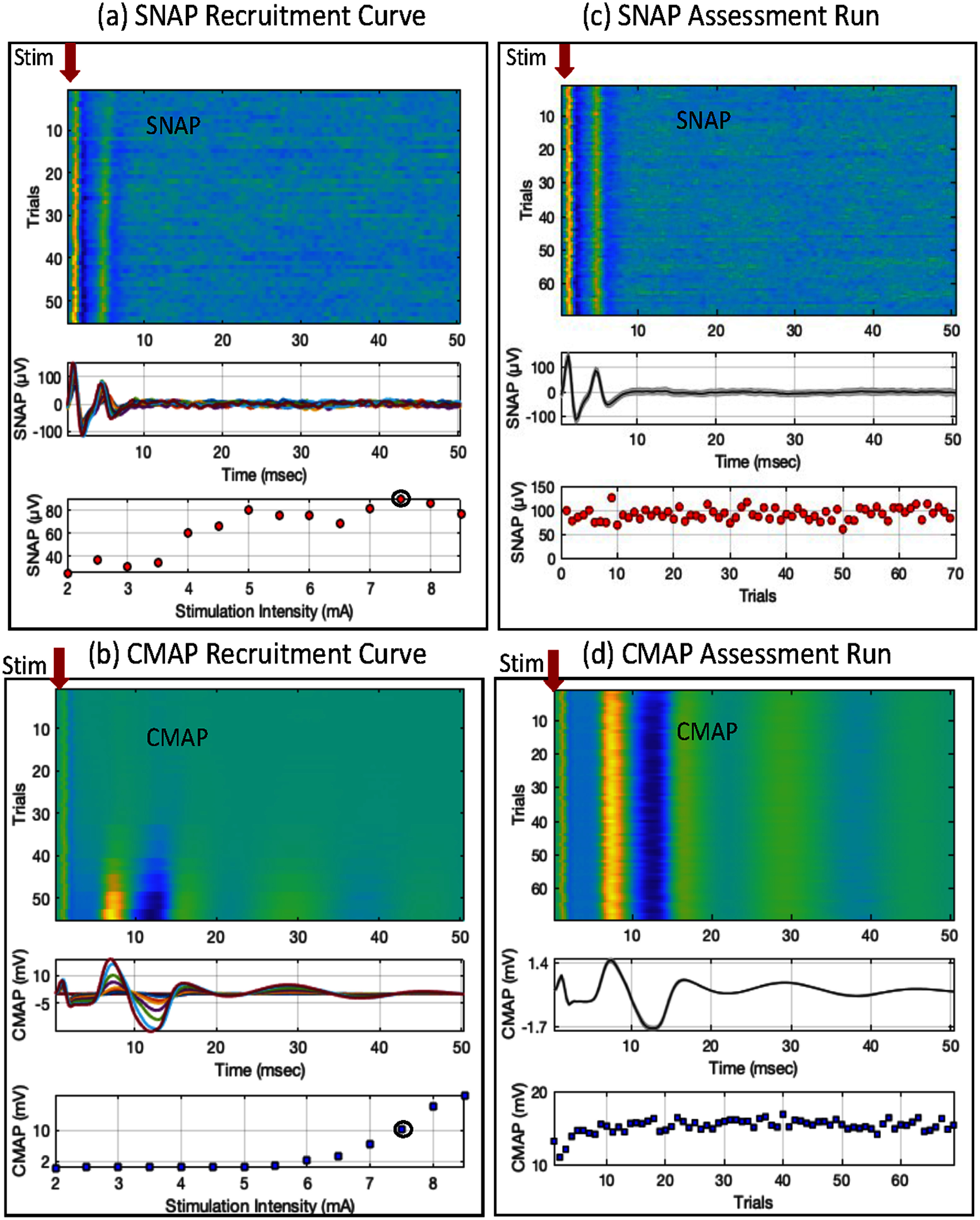
Sensory nerve action potential (SNAP) and compound muscle action potential (CMAP) from a representative participant during recruitment (left panels) and assessment run (right panels): (a) and (b) recruitment panels for SNAP and CMAP show all the trials as a heat map, recorded at increasing stimulus intensity; trial time courses averaged at each intensity; and the averaged peak response as a function of stimulus intensity. (c) and (d) Assessment runs panels for SNAP and CMAP show all trials as a heat map; averaged time course, and the peak response across trials. The target stimulation intensity used in the assessment runs is marked on the respective recruitment curves. Stimulus onset (at 0 ms) is marked by a red arrow.

#### 2.7.2. Single trial SEP analysis

The single trial SEP was analyzed with minimal post-processing in order to mimic the real time analysis pipeline. Therefore, only spatial and temporal filters were used with no further processing for trial denoising. For each trial the root mean square (RMS) value of the epoch 25–65 ms for P50 and 55–95 ms for N70 was obtained at the spatially filtered contralateral C3/C4 electrode. In addition, for each trial, the RMS of a corresponding background epoch (−50 to 0 ms) was also obtained. The RMS helps to overcome short temporal jitter across trials and captures most of the energy of the components of interest, as these mid latency responses are typically broad. The separability of each of these univariate SEP peak responses was compared relative to the background epochs using the ROC AUC. The AUC being non-parametric and invariant to non-linear transformations, provides a robust measure of SEP separability from background.

### 2.8. Statistical analysis

All analysis was performed using MATLAB 2020b (MathWorks, MA). The SNR of the SEP peaks was calculated as the ratio of the mean and standard deviation (Smith 1997). The spatial SNR with respect to the background EEG was assessed with the coefficient of determination (*r*^2^), calculated as the squared correlation coefficient between the measured responses (i.e. SEP responses from all trials along with corresponding background amplitudes) and corresponding distinct labels assigned to these sets of responses. As most data were not normal (Lilliefors test (Lilliefors 1967), *p* > 0.05), non-parametric tests were used. The Friedman Repeated measures test was used for the assessment of repeated measures, followed by Wilcoxon Signed Rank test with Holm multiple comparison correction. The effect size was estimated using the coefficient of concordance (Kendall’s (Tomczak and Tomczak 2014)) with the equation $w = {\text{ }}{\chi ^2}/n\left( {k - 1} \right)$, where ${\chi ^2}$ is the Friedman test statistic, $n$ is the sample size, and $k$ is the number of repeated measurements. The interpretation of Kendall’s $w$ was based on Cohen’s interpretation guidelines (Cohen 1977) of 0.1−<0.3 (small effect), 0.3−<0.5 (moderate effect), and ⩾0.5 (large effect). *P* values <0.05 were considered significant. Coefficient of variation (CV) was used as a standardized measure of sample dispersion around the mean value (Everitt and Skrondal 2010). It was calculated as the ratio of the standard deviation and the mean of the signal of interest. A small CV (<0.5) shows less variability. Intraclass correlation coefficient (ICC) was used to evaluate the test-retest reliability (Koo and Li 2016, Liljequist *et al* 2019) across two sessions. ICC estimates and their 95% confidence intervals were calculated using MATLAB 2020b, based on a single measurement, absolute agreement, 2-way mixed-effects model. The ICC values were interpreted as per the guidelines (Koo and Li 2016) as 0.5 (poor), 0.5–0.75 (moderate), 0.75–0.9 (good) and >0.9 (excellent) reliability. All numerical values in the text are shown as mean ± standard error, unless the data has outliers, where a median ± standard error is used instead, and stated in the text.

## 3. Results

In the group of twelve healthy participants, nine individuals received stimulation on the left hand and three on the right hand (due to a history of an injury on the left hand). We record the SEP at intensities guided by the SNAP at the third digit and the CMAP at the APB muscle—both reflecting reliable activation of median nerve pathways. We monitor the SNAP and CMAP in real time to maintain stable effective afferent excitation. The SEP P50 and N70 components were observed in all participants, at the contralateral sensorimotor region (i.e. C3 for right hand and C4 for left hand stimulation). The median nerve SNAP amplitude has been shown to be less affected by gender but reduces with age (50–79 age group has a smaller SNAP relative to younger groups) (Buschbacher 1999, Chen *et al* 2016a). Median nerve CMAP amplitude is known to be less affected by gender and height but reduces with age (Chen *et al* 2016a). We see similar variations in our dataset which had 4 people < 50 years of age. Their SNAP responses were observed to be generally larger than the rest of the group. However, since the study analysis is conducted within subjects, these variations should not impact the overall study outcomes.

SEP was measured while monitoring the effective afferent stimulation via SNAP and the CMAP, using the EPOCS platform. A representative trial-wise and corresponding averaged SEP response for left hand stimulation is shown in figure 2, at a pulse width of 1 ms. The average latency of the P50 and N70 across participants at this pulse width were 44.2 ± 1.7 ms and 80.6 ± 4.1 ms; and the peak amplitudes were 55.4 ± 7.0 a.u. and −66.9 ± 8.6 a.u.

The average SNR (measured with *r*^2^) was 0.12 for P50 and 0.24 for N70. The SNAP and CMAP were observed in all participants, albeit quite noisy and unclear in one participant, whose nerve response data was removed from the SNAP and CMAP analysis. The SNAP and CMAP recruitment curve and assessment data from a representative participant (same as in figure 2) is shown in figure 3 (at a pulse width of 1 ms). The SNAP peak was observed at an average latency of 3.8 ± 0.1 ms and the CMAP peak was observed at an average latency of 6.0 ± 0.2 ms, across participants, in line with literature (Wróbel *et al* 2021).

**Figure 4.**
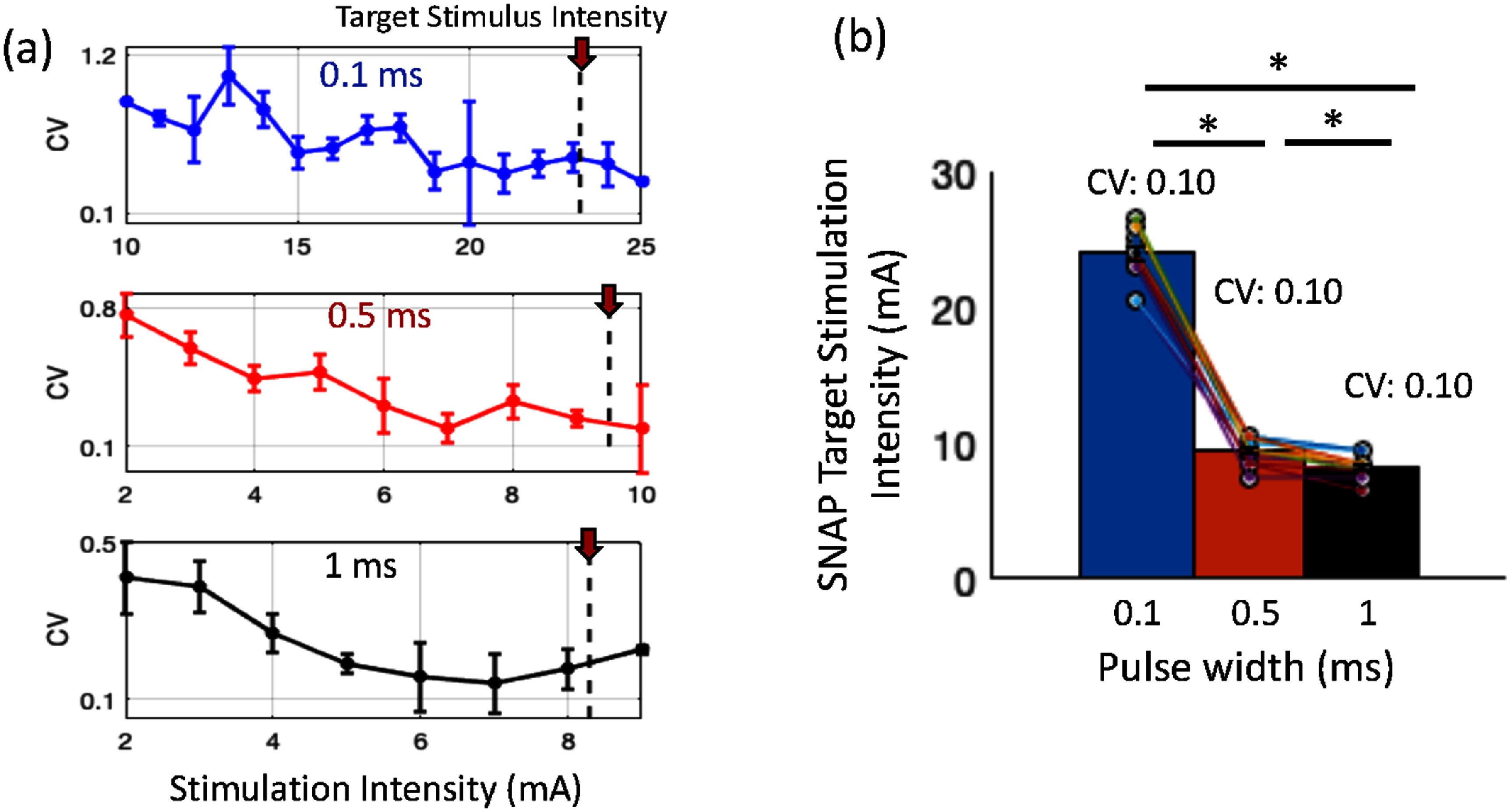
(a) Coefficient of variation (CV) of SNAP amplitude across stimulation intensities and pulse widths, across participants. CV for SNAP decreases with increase in stimulation intensity, at each pulse width. (b) Target stimulation intensity across pulse widths. Significant difference in target intensity is observed across pulse widths (Friedman repeated measures, p < 0.001). The absolute target intensities at each pulse width are similar (small CV) across participants.

The change in consistency of the SNAP response was measured by the CV (figure 4(a)). At all pulse widths, CV was found to be larger at low stimulation intensities which gradually decreased as stimulation intensity increased. Retrospectively, using the post-processed (filtered, denoised) signals, we find that this visually determined stimulus intensity is within 0.9 ± 0.2 mA of the intensity that first elicits a SNAP peak larger than the background (i.e. >median +3 × standard deviation) in 3 out of 4 trials. Since the stimulation intensity was determined to ensure consistent SNAP responses, further adjustments during the assessment run were expected to be minimal. Intensity changes were required in only 1–2 participants per pulse width (i.e. 2 participants for 0.1 ms, 2 for 0.5 ms, and 1 for 1 ms), occurring 2–3 times throughout their respective assessment run.

The absolute target stimulation intensities determined with the above criterion were similar across participants, at each pulse width (tabulated in table 1, and figure 4(b)), reflected by the small CV. Normalized target intensity is also shown in table 1, where the target intensity at each pulse width was normalized by the corresponding target intensity at 1 ms for each participant. The stimulation intensities were significantly different across pulse widths as expected (Friedman test of repeated measures: ${\chi ^2}$ = 23.53, *p* = 7.76 × 10^−06^, *w* = 1.0). The *post hoc* test showed a significant difference between all pairs of pulse widths (*p* < 0.05, corrected by Holm multiple comparison correction).

**Table 1.** Target stimulation intensity and its coefficient of variation across pulse widths, across participants.

Pulse width (ms)	0.1 ms	0.5 ms	1 ms
Target stimulation intensity (mA)	24.2 ± 0.6 mA	9.5 ± 0.3 mA	8.3 ± 0.2 mA
Coefficient of variation	0.10	0.10	0.10
Normalized target stimulation intensity	3.0 ± 0.1	1.1 ± 0.0	1.0 ± 0.0

### 3.1. SEP N70 increases with pulse width

**Figure 5.**
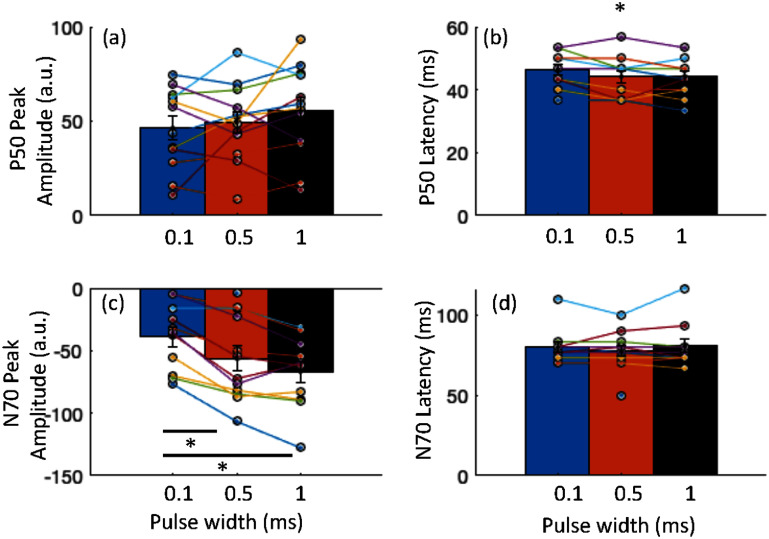
Somatosensory evoked potential N70 and P50 amplitude and latencies across participants. (a) and (c) P50 and N70 peak amplitudes at three pulse widths. N70 peak increases significantly with longer pulse width (Friedman repeated measures test, p = 0.0001; post hoc tests show significant difference between 0.1 and 0.5 ms (p = 0.001) and 0.1 and 1 ms pulse (p = 0.001)); (c) and (d) P50 and N70 latencies at the three pulse widths. P50 latency showed a small but significant change (p = 0.040) while N70 latency remained unchanged. Post hoc p-values were Holm corrected.

SEP P50 and N70 peaks were evaluated at three pulse widths −0.1 ms, 0.5 ms and 1 ms. The spatial distribution of these peaks (calculated with *r*^2^) was found to be centered at the sensorimotor region contralateral to the stimulated hand i.e. spatially filtered C3 for right hand stimulation and C4 for left hand stimulation. Figure 5 shows the *r*^2^ for the N70 (first row) and P50 (second row) responses averaged across all participants. For those who received right-hand stimulation, the topography has been mirrored for visualization.

**Figure 6.**
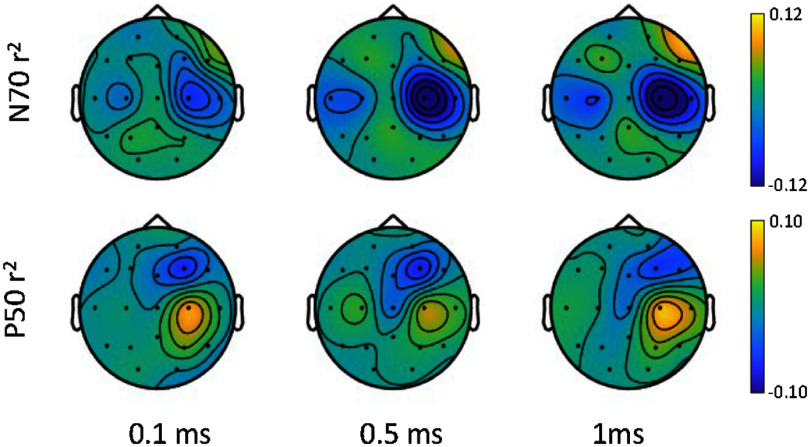
Coefficient of determination (r^2^) as a measure of spatial signal-to-noise ratio of the somatosensory evoked potential (SEP) components, across participants: (top row): spatial distribution of SEP N70 r^2^ at the three pulse widths; (bottom row): spatial distribution of SEP P50 r^2^ at the three pulse widths, across participants.

The average P50 and N70 peaks and latencies, across participants, are tabulated in table 2 and shown in figure 6. The SEP N70 peak amplitude across the three pulse widths was tested with Friedman analysis of variance for repeated measures, with pulse width as a factor. It showed that a change in pulse width significantly affected the SEP N70 peak amplitude (${\chi ^2}$ = 17.64, *p* = 0.0001, *w* = 0.80). Post hoc tests showed that the N70 elicited by 0.1 ms pulse width was significantly different (Holm corrected) than that elicited by 0.5 ms (*p* = 0.001,) and 1 ms (*p* = 0.001). Friedman analysis of variance on the P50 peak did not show a significant effect of pulse width (${\chi ^2}$ = 4.5, *p* = 0.105). The N70 latency did not show a significant change with pulse width (${\chi ^2}$= 0.24, *p* = 0.887), however, P50 showed a small but significant increase in latency (${\chi ^2}$= 6.41, *p* = 0.040, *w* = 0.27), however post hoc tests with Holm correction did not show a significance difference between the pairs (*p* > 0.05).

**Table 2.** Somatosensory evoked potential N70 and P50 amplitude and latencies at three pulse widths.

Pulse width	P50 amplitude (a.u.)	N70 amplitude (a.u.)	P50 latency (ms)	N70 latency (ms)
0.1 ms	46.4 ± 6.2	−39.0 ± 7.9	46.4 ± 1.5	80.0 ± 3.3
0.5 ms	49.3 ± 5.9	−55.1 ± 9.8	44.2 ± 1.8	77.5 ± 3.5
1 ms	55.4 ± 7.0	−66.9 ± 8.6	44.2 ± 1.7	80.6 ± 4.1

### 3.2. N70 SNR improves at longer pulse width

**Figure 7.**
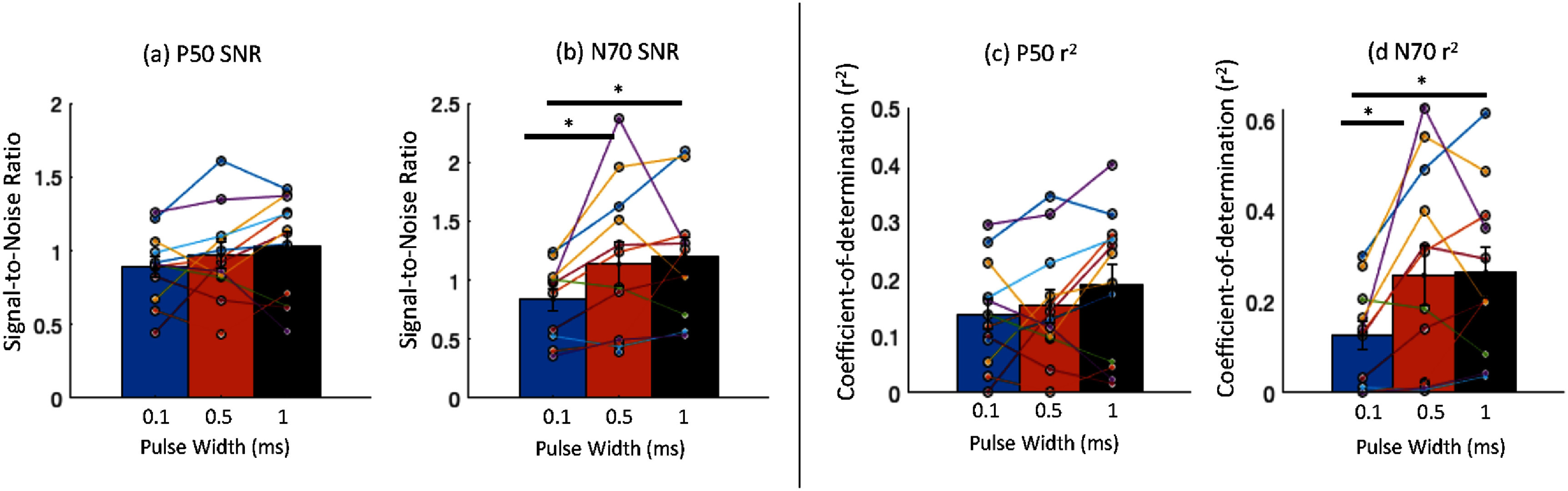
Signal-to-noise ratio for the somatosensory evoked potential components P50 and N70: (a) and (b) P50 and N70 SNR at three pulse widths. N70 SNR increases significantly with pulse width (Friedman repeated measures test, p = 0.02; post hoc tests show significant difference (Holm corrected) between 0.1 ms and 0.5 ms (p = 0.010) and between 0.1 and 1 ms pulse (p = 0.010)). (c) and (d) P50 and N70 SNR measured as the coefficient of determination (r^2^), with respect to the background EEG. N70 r^2^ increases significantly with pulse width (Friedman test, p = 0.01; post hoc tests show significant difference between 0.1 ms and 0.5 ms (p = 0.007) and between 0.1 and 1 ms (p = 0.007).

The absolute SNR for N70 peak increased from an average ± SE of 0.84 ± 0.09 (0.1 ms), to 1.13 ± 0.19 (0.5 ms), and 1.20 ± 0.16 (1 ms) (shown in figure 7(b)). A Friedman test showed this to be a significant change (${\chi ^2}$ = 7.82, *p* = 0.02, *w* = 0.35). Post hoc tests showed a significant difference (Holm corrected) between 0.1 ms and 0.5 ms (*p* = 0.010) and 0.1 and 1 ms (*p* = 0.010). The average percent change in SNR N70 peak, relative to that at 0.1 ms pulse width, was 39.7 ± 12.2% (at 0.5 ms) and 52.5 ± 19.0% (at 1 ms).

The absolute SNR for P50 peak was on average 0.90 ± 0.07 (0.1 ms), 0.97 ± 0.09 (0.5 ms), to 1.00 ± 0.10 (1 ms) (shown in figure 7(a)). However, this change was not significant (${\chi ^2}$ = 3.5, *p* = 0.174). The percent change in SNR for P50 peak relative to 0.1 ms was 12.7 ± 11.2% (at 0.5 ms), and 23.7 ± 16.3% (at 1 ms).

In addition, we calculate the coefficient of determination (*r*^2^), which represents the amount of signal feature variance explained by the stimuli response and background activity. The average *r*^2^ for N70 peak at contralateral electrode showed a significant increase with pulse width (shown in figures 7(c) and (d)): 0.13 ± 0.03 (at 0.1 ms), 0.25 ± 0.06 (at 0.5 ms) and 0.26 ± 0.05 (at 1 ms); ${\chi ^2}$= 8.91, *p* = 0.012, *w* = 0.40; post hoc test showed a significant difference (Holm corrected) between 0.1 and 0.5 ms (*p* = 0.007) and between 0.1 and 1 ms (*p* = 0.007). The average *r*^2^ for P50 peak also showed a small but non-significant (${\chi ^2}$= 3.5, *p* = 0.174) increase with pulse width 0.14 ± 0.03 (at 0.1 ms), 0.15 ± 0.03 (at 0.5 ms) and 0.19 ± 0.04 (at 1 ms).

### 3.3. Increased afferent input at longer pulse width at the target intensity

According to the equation (1), the charge delivered at 1 ms pulse duration is on average 0.0083 (Amp) × 0.001 (sec) = 8.30 × 10^−06^ C. This amount of charge adequately elicited discernable and consistent SNAP and CMAP responses along with robust SEP responses, with little discomfort. At 0.5 ms pulse width, a smaller average charge of 4.75 × 10^−06^ C was required, and at 0.1 ms, a much smaller charge of 2.32 × 10^−06^ C was required to elicit a similarly robust and discernable SNAP and CMAP. However, the SNAP amplitude was significantly smaller at these lower pulse widths–0.5 ms (75.01 ± 4.3%) and 0.1 ms (30.02 ± 3.4%) relative to the amplitude at 1 ms (Friedman test, ${\chi ^2}$= 20.18, *p* = 4.145 × 10^−05^, *w* = 0.91). The corresponding SEP N70 amplitude and its SNR was also significantly smaller at 0.1 ms pulse width.

### 3.4. Session to session variability decreases at 0.5 ms pulse width

We evaluate the test-retest reliability of the N70 and P50 peak and latencies at the three pulse widths, using ICC, with data from ten participants who participated in two sessions. In three participants the data in the additional session was available for only 0.1 and 0.5 ms pulse widths. The ICC (2,1) was used, with a 2-way mixed model, single measurement, absolute agreement. The ICC scores and the *p*-values are tabulated in table 3. The ICC for N70 and P50 peak amplitudes and latencies were lower at 0.1 ms pulse width and improved for both N70 and P50 at the longer pulse width of 0.5 ms. The stimulation intensity differed between the sessions by a mean of 1.5 ± 0.2 mA.

**Table 3.** Intraclass correlation coefficient (ICC) for test-retest reliability across sessions, across participants.

	Pulse width (0.1 ms)	Pulse width (0.5 ms)	Pulse width (1 ms)
	*n* = 10	*n* = 10	*n* = 7
	ICC (2,1), *p*-value	ICC (2,1), *p*-value	ICC (2,1), *p*-value
N70 amplitude	0.68, 0.012	0.76, 0.004	0.68, 0.019
P50 amplitude	0.36, 0.143	0.84, 0.0003	0.57, 0.066

N70 latency	0.28, 0.220	0.76, 0.004	0.91, 0.0006
P50 latency	0.56, 0.034	0.70, 0.01	0.68, 0.021

Note: ICC Scores- Poor (0.5), Moderate (0.5–0.75), Good (0.75–0.9), Excellent (> 0.9)

### 3.5. Separability of single trial N70 increases at higher pulse width

**Figure 8.**
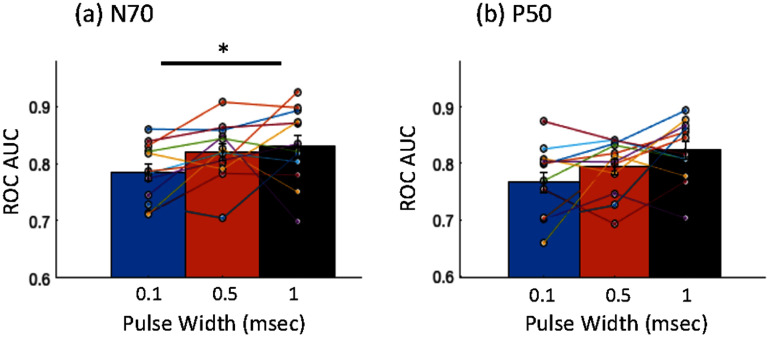
Separability of single trial somatosensory evoked potential (SEP) components N70 and P50 at pulse widths of 0.1 ms, 0.5 ms and 1 ms. Receiver operating characteristics area under the curve (ROC AUC) is used to quantify the separability of SEPs from background activity. N70 shows a significant difference across pulse widths, tested with Friedman repeated measures test (p = 0.017). Post hoc test shows significance difference between 0.1 and 1 ms pulse widths (p = 0.007).

To quantify the ability to distinguish SEPs from background noise in single trials, we evaluate the ROC AUC for the P50 and N70 relative to background epochs. These were obtained for the spatially filtered contralateral electrode (C3 or C4). The mean and standard error AUC for N70 component across participants was 0.78 ± 0.01, 0.82 ± 0.01, and 0.83 ± 0.02 for 0.1, 0.5 and 1 ms respectively. The corresponding mean and standard error for P50 AUC were 0.77 ± 0.02, 0.79 ± 0.01 and 0.82 ± 0.01, respectively, as shown in figure 8. The Friedman repeated measures test showed that the N70 AUC were significantly different across the pulse widths (${\chi ^2}$ = 8.17, *p* = 0.017, *w* = 0.34). Post hoc tests showed a significant difference (Holm corrected) for N70 AUC between 0.1 ms and 1 ms pulse widths (*p* = 0.007). The P50 AUC had a similar trend i.e. a higher AUC at higher pulse width, but did not show a significant difference with repeated measures test between the pulse widths (${\chi ^2}$= 5.17, *p* = 0.075).

### 3.6. Pilot SEP measurement in individual with iSCI

**Figure 9.**
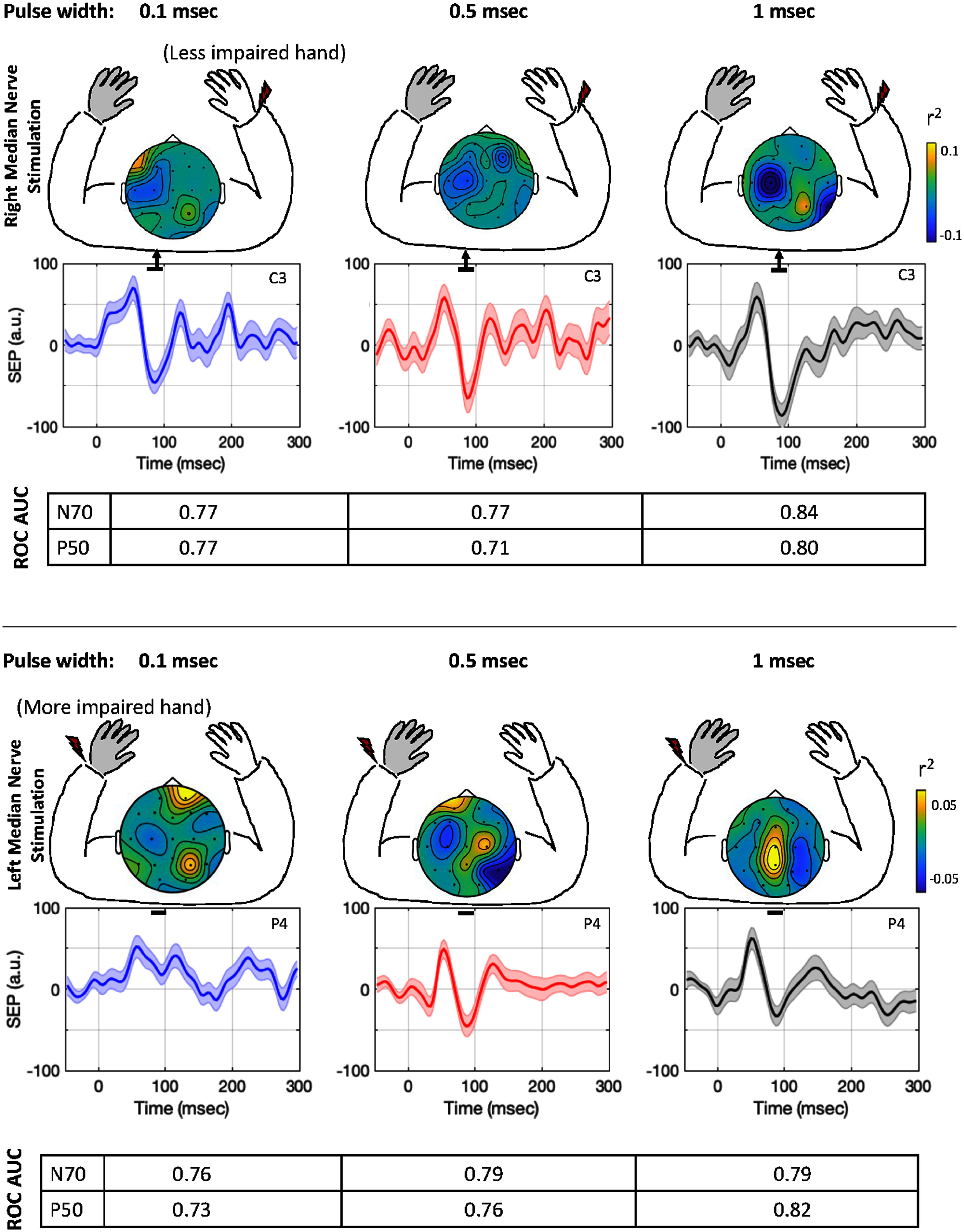
Somatosensory evoked responses (SEPs) in individual with incomplete spinal cord injury: Top panel shows the SEPs at the three pulse widths (0.1, 0.5 and 1 ms) when the right hand (less affected) median nerve is stimulated. The morphology and topography is similar to that observed in healthy people. At longer pulse width the SEP N70 magnitude is observed to be larger, with a higher spatial specificity, and a higher single trial separability (measured by the receiver operating curve area under the curve (ROC AUC)). Lower panel shows the SEPs elicited at P4, at the three pulse widths when the left hand (more affected) is stimulated, with a generally smaller N70 magnitude. The expected SEP morphology is observed at P4 at higher pulse widths. The spatial specificity and single trial separability is higher at longer pulse width.

The participant with iSCI mentioned marked sensory impairment in their left hand relative to their right hand, confirmed by the assisting physical therapist. Their SEP responses to right-hand (less affected) stimulation showed SEPs that had a topography and signal morphology like that observed in the healthy group (figures 2 and 5). The P50 and N70 components had a similar latency (for P50 and 86.5 ms for N70, at all three pulse widths), as the healthy group. The SEP response elicited by the left-hand (more affected) stimulation showed an atypical SEP response. As shown in figure 9, the N70 component was smaller in magnitude, and spatially prominent at the parietal cortical area (electrode P4), with an absence of a distinguishable response at the C4 electrode that was generally observed in the healthy group and for this individual’s less affected right-hand stimulation.

The SNR of N70 for the less affected right hand showed an increase from 1.0 at 0.1 ms and 0.5 ms pulse widths, to 1.3 at 1 ms pulse width, more similar to the SNR observed in healthy participants. The SNR for the more affected left hand, at P4, was observed to be much smaller, 0.09 for 0.1 ms pulse width, with an increase to 0.75 at 0.5 ms, and 0.60 at 1 ms pulse width. The single trial SEP analysis showed the feasibility of extracting SEPs relative to background activity at the proposed stimulation parameters (figure 9). Participant showed an increase in SEP separability for both the N70 and P50 at a longer pulse width relative to 0.1 ms, at the more affected hand.

A similar trend was observed for the less affected hand, except for the P50 at 0.5 ms that showed degradation. Assessment across more participants with iSCI would be useful to determine statistical differences between healthy people and those with iSCI.

## 4. Discussion

In this study we evaluate a few methodological approaches to improve the measurement of single trial SEP responses specific to electrical stimulation at the median nerve. The overarching aim of these optimizations is to be able to extract a somatosensory response in real time, distinguishable from background fluctuations. These include (a) optimization of stimulation parameters, (b) methods to maintain stable effective afferent excitation, and (c) recommendations for a robust SEP acquisition and preprocessing to maximize single trial SNR.

### 4.1. Optimizing stimulation parameters

SEP characteristics are strongly shaped by stimulation parameter choices, each with distinct advantages and drawbacks. Increasing stimulation intensity at short pulse widths can enhance SEP magnitude and SNR by recruiting a greater population of A*β* fibers. However, supramaximal intensities also activate higher-threshold A*δ* fibers, which transmit pain, crude touch, and temperature via the spinothalamic tract. Their recruitment can introduce discomfort, reduce tolerance for longer recordings, and confound cortical responses with pain-related activity. Excessive intensity may also spread to the neighboring ulnar nerve, further contaminating cortical signals. Beyond a saturation point, SEP amplitude no longer increases with intensity and may even attenuate due to discomfort or movement-related gating effects (Papakostopoulos *et al* 1975, Jones 1981, Valeriani *et al* 1998). An alternative is to manipulate pulse width. According to strength–duration relationships (Irnich 1980, Nelson and Hunt 1981), longer pulse widths reduce the current required to activate sensory fibers, reflecting their lower rheobase and longer time constant relative to motor fibers (Veale *et al* 1973, Panizza *et al* 1992). Thus, pulse widths ⩾0.5 ms preferentially recruit afferents over motor fibers (Kiernan *et al* 1996, Mogyoros *et al* 1996, Panizza *et al* 1998), limiting direct muscle responses and movement artifacts. Longer pulse widths also enhance spatial specificity, allowing activation of deeper nerves (Doucet *et al* 2012) while minimizing twitch-related gating effects (Onishi *et al* 1991). Pulse widths upto 1 ms have been used in multiple studies of peripheral stimulation (Gupta *et al* 2007, Thompson *et al* 2009, 2013, Carrico *et al* 2016, Conforto *et al* 2018, van ‘t Veld *et al* 2021, Kato *et al* 2023, Gupta *et al* 2025a, 2025b), with fewer studies assessing the SEPs evoked at these settings (Kato *et al* 2023, Gupta *et al* 2025a, 2025b).

Stimulation frequency also affects these trade-offs. Higher frequencies (such as 2 Hz) paired with suprathreshold, or long-duration pulses can exacerbate discomfort and gating (Gupta *et al* 2025a). In contrast, low-frequency stimulation (⩽1 Hz, with jitter) minimizes habituation and allows stable afferent recruitment with less contamination from motor-related activity.

### 4.2. Maintaining stable effective afferent stimulation

In peripheral nerve stimulation, the delivered current alone may not reliably predict the degree of afferent activation. Effective afferent excitation—the actual neural input reaching the sensory cortex—can fluctuate due to several physiological and experimental factors, including nerve temperature, joint angle, muscle tone, and local impedance variability caused by skin moisture, gel drying, or electrode shifts. These fluctuations contribute to trial-to-trial variability in SEP features and are particularly difficult to manage across sessions. To minimize such variability, our prior work (Gupta *et al* 2025a, 2025b) explored the use of M-wave and H-reflex monitoring to estimate effective stimulation levels in lower limb studies. While this strategy proved useful for nerves such as the tibial nerve, it is less applicable for more proximal nerves like the median nerve, where the H-reflex can become difficult to isolate due to temporal overlap with the CMAP and sensitivity to muscle contraction, posture, electrode positions, etc (Gupta *et al* 2021, Mercan and Kuruoğlu 2024). In this study, we instead use the SNAP recorded at the digit and the CMAP recorded from the APB muscle as substitutes for ensuring a consistent afferent pathway activation. Stimulation intensities that evoke a maximal SNAP have been explored before for obtaining a consistent SNAP recording (Nashed *et al* 2009). A stimulus intensity of 80% of the maximal SNAP was also shown to elicit saturated early SEP responses (Fukuda *et al* 2007). We found that a stimulation intensity—determined by the elicited SNAP and CMAP exceeding three standard deviations in 3 out of 4 trials—is sufficient to maintain the nerve potential within 20% of the target response.

Importantly, this method also circumvents the need for subjective motor or sensory thresholding—typically based on visible muscle twitch or verbal feedback—which is often unreliable. Such thresholds vary with individual characteristics (e.g. age, fatigue, body composition) and are even more challenging to determine in people with brain or spinal injuries, in whom localized sensory perception may be severely impaired and motor responses may be delayed or diminished. Hence, objective physiological markers like SNAP and CMAP provide a more robust, form of controlling afferent stimulation across sessions.

### 4.3. Effect of pulse width on SEP SNR, test-retest reliability, and single-trial detection

In healthy participants, using the proposed setup, both the P50 and N70 components were consistently elicited across all tested pulse widths, with minimal discomfort. The topography of the response was consistent across people, specific to the centro-parietal region contralateral to the stimulated hand, as expected. The N70 amplitude increased significantly with longer pulse widths, whereas the P50 amplitude remained stable. Importantly, SNR also improved significantly with increasing pulse width, with gains of 40% at 0.5 ms and 52% at 1 ms relative to 0.1 ms stimulation. The stability of the P50 and the amplification of the N70 suggest that longer pulse widths allow stronger, more synchronized cortical activation of the somatosensory cortex, while still avoiding excessive co-activation of motor fibers that can introduce artifacts or gating effects. The SEP test–retest reliability was found to be highest at 0.5 ms (ICC = 0.70–0.84, *p* < 0.05), indicating more consistent N70 and P50 responses at this intermediate pulse width. In single-trial analysis, the separability of the N70 was significantly enhanced at 1 ms (AUC = 0.83, *p* = 0.017), suggesting that longer pulse widths may better support real-time SEP detection.

Together, these findings highlight complementary trade-offs: 0.5 ms stimulation appears optimal for reliability and reproducibility, whereas 1 ms maximizes SEP magnitude and single-trial separability. These results are particularly relevant for real-time applications, such as operant conditioning and closed-loop BCIs, where robust single-trial detection and cross-session stability are essential.

### 4.4. Selection of an optimal recording system

Intra- and inter-session SEP variability can be influenced by the quality of the EEG recording system. Our study demonstrates the use of a dry active EEG headset, and Laplacian spatial filters for spatial processing, for robust single trial SEP acquisition. In single trial real time applications, as the post hoc averaging of trials, and advanced denoising options are limited, the selection of EEG system and the EEG pre-processing strategies (such as the temporal and spatial filters) need careful planning. Systems with active electrodes (i.e. with built-in preamplifiers at the electrode sites) can significantly reduce cable-related artifacts and environmental interference by boosting signal strength prior to transmission; wireless systems further eliminate movement-induced noise associated with tethered cables, which is particularly useful in protocols involving arm or body movements; high common-mode rejection ratio can attenuate environmental electrical noise, especially in less shielded settings. Errors introduced by inconsistent electrode placement across sessions can further degrade SEP signal reproducibility; thus, designs that allow replicating the electrode positions robustly on the scalp can minimize electrode variation errors across sessions.

### 4.5. Feasibility of single trial SEP measurement in spinal cord injury

SEP measurement was feasible in the individual with spinal cord injury using the stimulation parameters evaluated in this study. SEPs were elicited from both the less and the more impaired upper limb. On the less impaired side, the P50 and N70 components closely resembled those of the healthy group in terms of spatial distribution (contralateral centro-parietal, C3 electrode), latency, morphology, and amplitude. The N70 magnitude increased with longer pulse widths while latency remained stable, consistent with observations in healthy participants. The P50 component was comparatively stable across pulse widths, again resembling the pattern seen in healthy people. Single-trial SEP separability (ROC AUC of 0.77–0.84) on the less impaired side was also comparable to the healthy group, particularly at longer pulse widths, though somewhat reduced at shorter pulse widths.

On the more impaired side, the SEPs were less typical across all pulse widths. While the latencies of the P50 and N70 components were comparable to those observed on the less impaired side and in healthy participants, the spatial distribution was shifted posteriorly toward parietal regions, with reduced activation over C4–contrary to the healthy pattern. The N70 amplitude was markedly smaller relative to both the less impaired side and healthy participants, whereas the P50 peak was less affected. Notably, the N70 waveform became more typical in morphology at longer pulse widths despite its reduced amplitude, while at 0.1 ms it appeared noticeably distorted. The single-trial SEP separability was reasonable (ROC AUC of 0.76–0.79), though slightly lower than in the healthy group, despite the smaller response magnitude.

This pilot test highlights the value of optimized stimulation parameters for improving SEP detectability in people with iSCI. The observed asymmetry further suggests that SEPs may provide a useful marker of impaired afferent conduction and cortical sensory processing in iSCI, although confirmation in a larger cohort is needed. These findings support the potential for SEP-based BCI applications in iSCI, indicating that even in impaired pathways, optimized parameters can evoke measurable SEP responses suitable for real time applications.

### 4.6. Limitations and future applications

These optimizations establish a framework for BCI applications in neurorehabilitation that leverage cortical afferent responses. There is a need to assess the feasibility of these optimizations in older people, as the nerve and cortical responses can be qualitatively different. In this study we did not separately assess the effect of handedness which can be useful to assess as a factor.

Future studies will evaluate the feasibility of an SEP-based BCI by examining immediate, short-term effects on SEPs, followed by efficacy testing across repeated training sessions. The real time selection of stimulation intensity will be incorporated as an automated algorithmic selection in the EPOCS system. The development of a portable system that integrates peripheral stimulation and response monitoring with a synchronized BCI platform, may facilitate translation to home-based rehabilitation. Beyond translational applications, these developments can also advance mechanistic research into the somatosensory contributions to movement recovery and inform the design of novel therapeutic strategies.

## 5. Conclusion

The study describes and evaluates methodological optimizations for eliciting robust median nerve SEPs—both averaged and single-trial—using noninvasive EEG. These optimizations include the combination of a longer pulse width with lower stimulation frequency, and a higher stimulation intensity, guided by the SNAPs and direct muscle responses. It also emphasizes active monitoring of effective afferent excitation and the use of a robust SEP recording system.

The results demonstrated improvements in N70 amplitude, SEP SNR, test–retest reliability, and single-trial detectability, supporting the use of longer pulse widths (0.5–1 ms) with higher stimulation intensity and low frequency (0.5 Hz) as an effective strategy to enhance SEP robustness. The pilot test in an individual with spinal cord injury illustrates the feasibility of applying these optimizations in people with spinal injuries, though larger studies are needed.

These findings are relevant not only for understanding sensory processing mechanisms but also for advancing translational applications of SEP-based BCIs. Such approaches may support rehabilitation research and development in spinal cord and other brain injuries, where reliable SEP measurement and feedback can contribute to novel therapeutic strategies.

## Data Availability

The data cannot be made publicly available upon publication due to legal restrictions preventing unrestricted public distribution. The data that support the findings of this study are available upon reasonable request from the authors.

## CRediT

Disha Gupta: Conceptualization, methodology, software, data collection, analysis, results interpretation, writing- original draft preparation.

Jodi Brangaccio: Recruitment, data collection.

N. Jeremy Hill: Conceptualization, hardware/software, results interpretation, writing-reviewing.
